# The features and processes underpinning high‐quality data generation in participatory research and engagement activities

**DOI:** 10.1111/2041-210X.13746

**Published:** 2021-10-31

**Authors:** Phoebe R. Maund, Jacob W. Bentley, Gail E. Austen, Katherine N. Irvine, Robert Fish, Martin Dallimer, Zoe G. Davies

**Affiliations:** ^1^ Durrell Institute of Conservation and Ecology (DICE) School of Anthropology and Conservation University of Kent Canterbury UK; ^2^ UN Environment Programme World Conservation Monitoring Centre (UNEP‐WCMC) Cambridge UK; ^3^ Social, Economic and Geographic Sciences Department James Hutton Institute Aberdeen UK; ^4^ Sustainability Research Institute School of Earth and Environment University of Leeds Leeds UK

**Keywords:** conservation, environment, interdisciplinary, policy, practice, qualitative, social science, transdisciplinary

## Abstract

1. Participatory approaches are widely used by researchers to gather data and insight about how the environment is perceived, valued and used. The participatory activities may be creating information as part of curiosity‐driven blue‐skies research or to inform policy/practise decision‐making.
2. The quality and usability of data derived from participatory approaches are heavily influenced by how activities are conducted. We share a set of features and processes that underpin the generation of high‐quality data, based on our collective experience of developing and undertaking participatory activities with an environmental and conservation focus.
3. We propose four general features: (a) Depth and breadth of engagement; (b) robustness of the approach; (c) allowing space for surprises; (d) usability across contexts. We also provide a practical toolbox of processes, and associated facilitation techniques, which can be employed to maximise participant engagement and generate quality data.
4. The features and processes are a practical guide for project leaders/teams to consider in the context of their work, rather than a set of inflexible rules. They should be relevant regardless of the participatory methods used, or the research, policy or practice setting being addressed.

## 1 INTRODUCTION

Participatory approaches to data collection and knowledge production are widely used to deepen our understanding of how humans perceive, value and use the environment (Bennett et al., 2016). They can be defined as ‘*a relational process through which new knowledge is produced collectively rather than by an individual on their own. The purpose of that new knowledge is to bring about some form of change or action, whilst the process of doing so is a continual one of learning, reflection and action*’ (Abma et al., 2017). Participatory approaches are used globally, across the low‐, middle‐ and high‐income countries. Whilst the language used to describe these approaches can be diverse, reflecting their varied origins and contexts of use, they all involve creating data from the vantage point of individuals and communities. This can either be via direct interaction or through the formal groups and organisations that represent them. The purpose of participatory activities is to ascertain participant knowledge, insight, experiences and values in ways that could not be anticipated and identified by other modes of data collection, whether that is to reveal perspectives as part of curiosity‐driven blue‐skies research or to inform issues of a more applied policy/practice nature (Mukherjee et al., 2018).

Critically, the quality and usability of the data from participatory approaches can be heavily influenced by the way the activities are conducted (Young et al., 2018). Successful participatory approaches are predominately assessed by their legitimacy, defined by the fairness of the activities and whether they consider the full array of values, concerns and perspectives (Cash et al., 2003). However, legitimacy can be unduly influenced. For instance, by mistrust between the people involved, uneven power dynamics that can negatively influence the direction of the conversation and coerce particular opinions or insufficient time and resources that may limit the inclusion of some types of participants (Díez et al., 2015).

**FIGURE 1:**
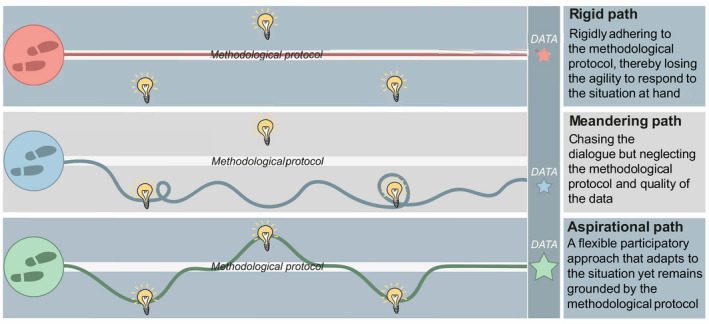
Conducting participatory activities. All three paths are possible ways to implement participatory activities, yet each will lead to different data and interpretation. To maximise the data quality, it is important to be adaptable and take advantage of opportunities to learn new information, whilst remaining grounded in a well‐conceived methodological protocol

The way in which the project leader/team plan, manage and deliver participatory activities, and the dialogue that occurs during them, will result in data of varying quality and interpretability (Figure 1). Here, we define quality data as those that have been generated via a robust and transparent process that enable the project leader/team to provide insights into the research question or policy/practice issue under investigation. Thus, quality data are both a condition of the process, as well as an outcome of it. If the project leader/team follow their methodological protocol too rigidly, they may lose the ability to take advantage of the richness of information that participants are willing to contribute. Conversely, undertaking a participatory activity without a well‐conceived methodological protocol could lead to unstructured and meandering discussions that may compromise the generation of relevant or complete data. Overall, it is important to strike a balance that nurtures productive and useful dialogue, whilst recognising that some participatory processes are methodologically more flexible than others. For example, a discussion group is a semi‐structured technique, whereas a focus group is a highly structured group interview (Payne & Payne, 2004). Equally, many widely used participatory approaches, such as participatory rural appraisal, encompass a variety of techniques that are adapted to suit the context (Chevalier & Buckles, 2019). With the growing popularity and application of participatory approaches, project leaders/teams should have an understanding of best practices they need to employ to minimise the risks associated with producing outputs that do not address the guiding purpose of the activity (Martin, 2019).

Participatory activities are made up of many junctures that project leaders/teams should adapt and respond to if the quality of the emerging data is to be maximised. Here, we draw on our collective transdisciplinary experience of developing and conducting participatory activities to share a set of features that underpin the generation of high‐quality data, along with the processes that support them. These should act as a practical guide for project leaders/teams to consider in the context of their work, rather than a set of inflexible rules. They should be relevant regardless of the participatory methods used, or the research, policy or practice setting being addressed.

## 2 THE FOUR FEATURES FOR GENERATING HIGH‐QUALITY DATA

**FIGURE 2:**
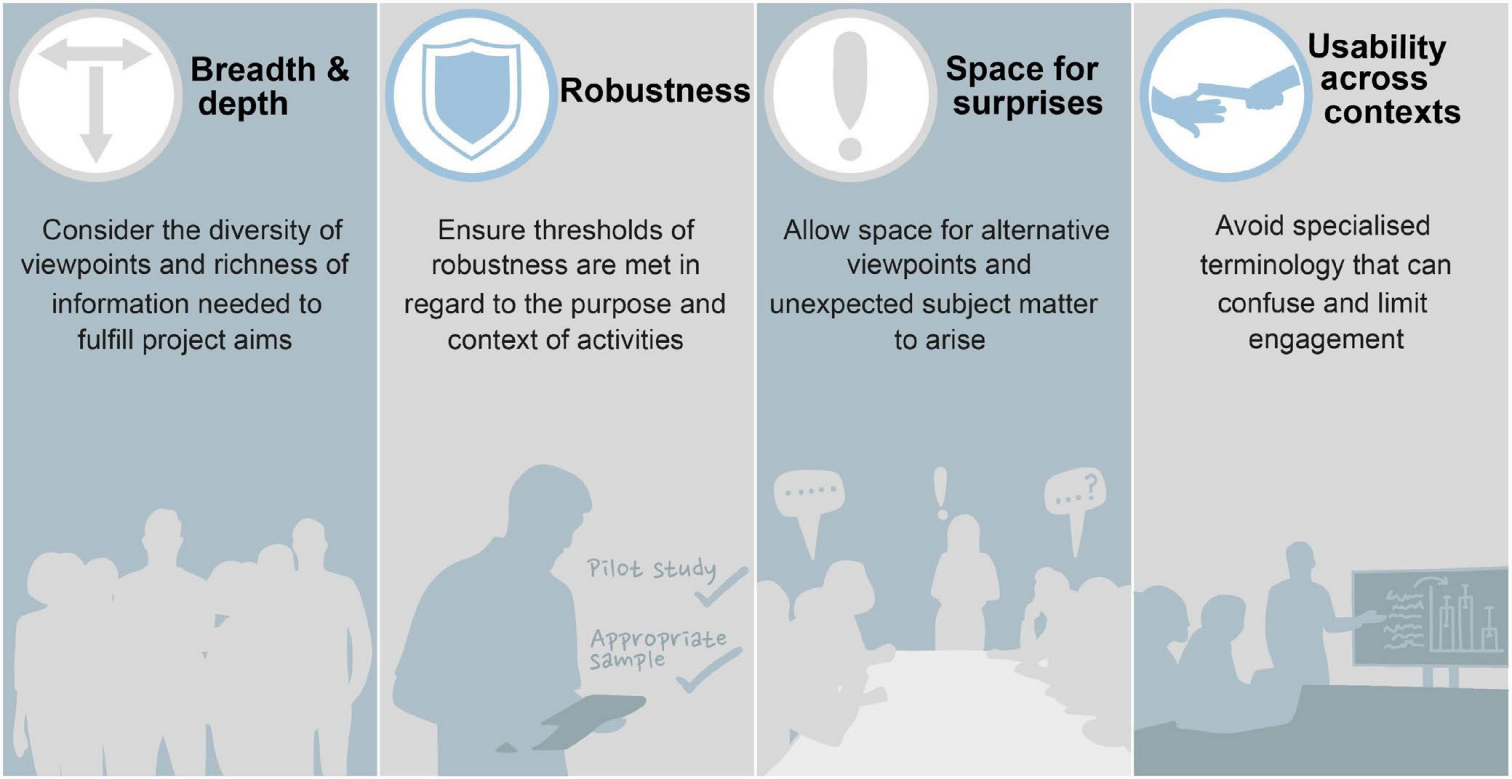
The four features for generating high‐quality data during participatory activities. These features help ensure the collection of high‐quality data that can be used for research purposes and/or to inform policy/practise decision‐making

We propose four general features as cornerstones of high‐quality data collection from participatory activities. Specifically, project leaders/teams need to consider: (a) Depth and breadth of engagement; (b) robustness of the approach; (c) allowing space for surprises; (d) usability across contexts (Figure 2).

### 2.1 Breadth and depth

Breadth (narrow to broad) and depth (shallow to deep) refer to the diversity of viewpoints and the richness of data required from a participatory activity (Table 1). Narrow participatory activities may only require a small and targeted group of participants and their viewpoints (Case Study 1). At the other end of the spectrum, broad activities are about garnering the range of viewpoints by representing the diversity of knowledge, insight, experiences and/or values across the participant cohort. For instance, this may require drawing together individuals from across large geographic distances, different cultural or sociodemographic backgrounds or with varying levels of expertise (Case Study 2). However, encapsulating breadth does not necessarily equate to a large number of participants. A balance also needs to be struck between representing and exhausting perspectives, and the associated need to explore underlying complexity and reasoning, which influences the depth of the participatory activity. Indeed, project leaders/teams may only require straightforward contributions, such as a simple statement of preference for one item in a selection. Thus, in practice, all modes of participatory activity (narrow and shallow, narrow and deep, board and shallow, broad and deep) can be consistent with good practice, depending on the situation at hand (Table 1).

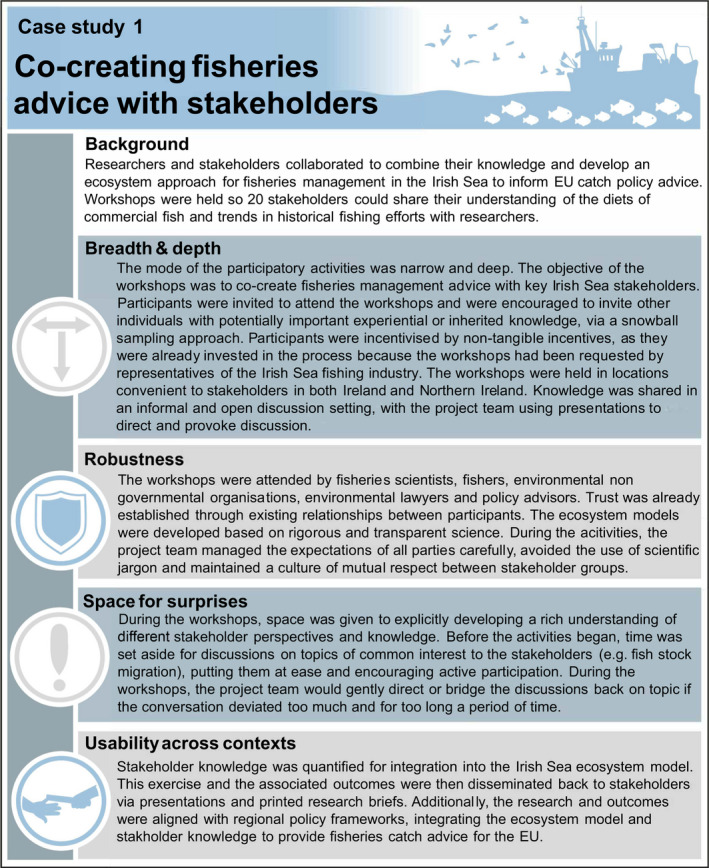

**TABLE 1:** Modes of participatory activity. Descriptions and examples of the depth and breadth of participatory activities: (i) narrow and shallow; (ii) narrow and deep; (iii) broad and shallow and (vi) broad and deep

Mode of participatory activity	Description	Example
Narrow and shallow	Small diversity of viewpoints providing straightforward information	Q‐methodology (Guenat et al., 2019). This activity involved a narrow group of stakeholders with an interest in urban greenspace management, from two small cities in western Ghana. It used a well‐defined mixed‐methods approach that allowed participants to reflect on, and influence, the outcomes of the research
Narrow and deep	Small diversity of viewpoints providing rich and nuanced information	Deliberative workshop (Kenter et al., 2016). This explored cultural ecosystem service values for proposed UK marine protected areas with a group of divers and recreational fishers in England and Scotland. The activity elicited the reasoning underpinning both individual and shared values
Broad and shallow	Large diversity of viewpoints providing straightforward information	Horizon scan using a modified Delphi approach (Goddard et al., 2021). This study was global in scope, engaging a wide range of experts from across disciplines and sectors aligned with robotics, urban planning and ecology. It sought to determine a list of challenges and opportunities associated with a pre‐determined specific topic
Broad and deep	Large diversity of viewpoints providing rich and nuanced information	Public dialogue (Fish & Saratsi, 2015). This detailed process, conducted in the UK, evaluated the ecosystems approach from a public perspective, involving contributions from local and national stakeholders from across sectors. The participants were both geographically and socially diverse. Participants were reconvened from regions into a national dialogue, revisiting findings from earlier in the process

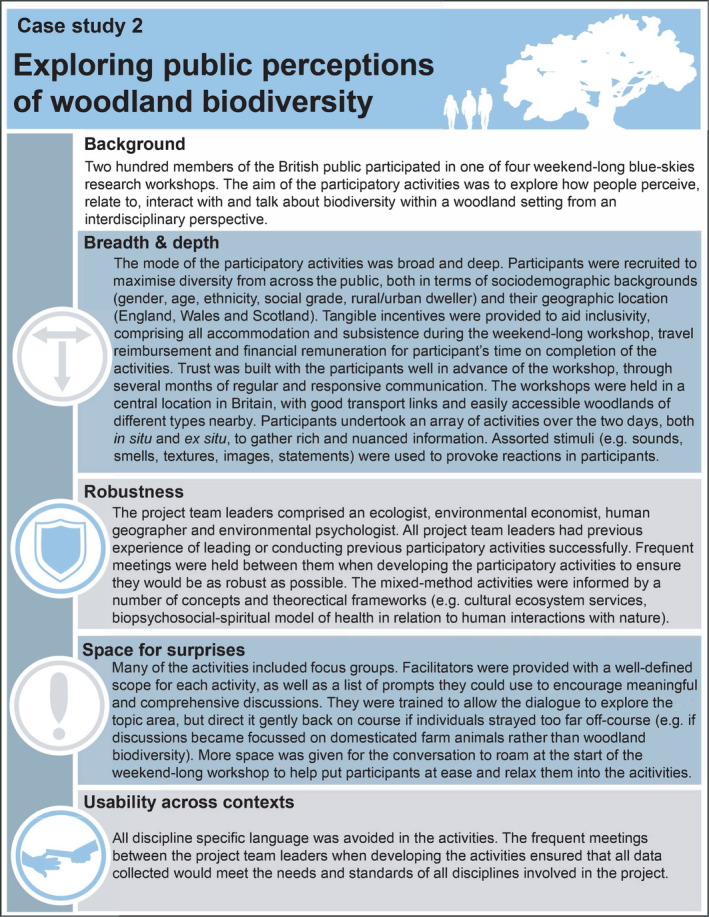

### 2.2 Robustness

Although the steps taken to produce robust data will vary depending on the purpose and type of participatory activity undertaken, all projects must produce outputs capable of withstanding scrutiny. Project leaders/teams must, therefore, understand the concepts and theoretical frameworks that will form the foundation of high‐quality empirical data collection. Additionally, we must be mindful of both participant biases (e.g. individuals conforming to viewpoints expressed by the rest of the cohort) and researcher biases (e.g. posing leading questions, making assumptions about participant responses based on one's own cultural context), ensuring these do not impact the integrity of the outputs. The project leader/team should evaluate whether they have the relevant skills to implement the participatory activities, including whether they can retain critical distance during the process, given their own knowledge, insight, experiences and values. Moreover, natural scientists, who are increasingly integrating social science techniques into their research, are sometimes critiqued for not possessing a solid understanding of the literature on participatory approaches and being inexperienced with the methods they apply (Martin, 2019). Likewise, not all social scientists are specialists in participatory approaches. In general, a collaboration between natural and social scientists with experience and expertise in conducting participatory activities will help to ensure robust data of the highest possible standard are produced.

### 2.3 Space for surprises

When designing participatory activities, space should be provided for unexpected data to arise. This serves two purposes. First, this helps to avoid the limitations and biases associated with assuming that the way participants communicate can be fully anticipated. Instead, participants should have opportunities to express what they believe is relevant, and highlight important perspectives and dimensions of topics that may not have been considered previously within the literature. Second, even when information from participants is not initially or obviously pertinent to the project aim, letting individuals pursue their own reasoning can often reveal hidden salience, whilst fostering an environment of trust and inclusion that can indirectly improve the quality of the data. Nonetheless, whilst it is valuable to allow space for the conversation to roam, it is important not to lose sight of the underlying methodological protocol (Figure 1). Deviations should enhance the study and the resulting data, not derail it. A balance can be achieved by having a clearly defined a priori scope and a list of topics for consideration. From this, the project leader/team may wish to develop a series of prompts to aid discussion. The project leader/team can continually refer back to this material throughout the participatory activities to ensure the conversation remains within scope, without the need for a prescriptive set of questions that could hamper the development of discussions.

### 2.4 Usability across contexts

It is important that the language the project leader/team uses, and the resulting data, insights and outputs, are capable of traversing contexts (e.g. between different disciplines, from research to policy/practise) so it can be as useful as possible. One component of this will be to reflect on how the collection of information could be managed to meet the needs of all disciplines involved in the project (e.g. thresholds of robustness, nature of the qualitative/quantitative information). Project leaders/teams should also reflect on how easily the data could be interpreted across contexts, both during the participatory activities and, subsequently, when the outcomes are communicated. For example, if the participatory activity is intended to influence policy, the structure of the policy framework should be understood, and project outputs should be directly relevant and interpretable in this context. Usability across contexts can also be hindered significantly by sector‐ or discipline‐specific jargon that fails to resonate with participants and/or project team members, limiting their ability to be fully immersed in the activities. This may reduce the quality of information generated through the activities. Additionally, jargon can limit the accessibility and use of project outputs.

## 3 PROCESSES

**FIGURE 3:**
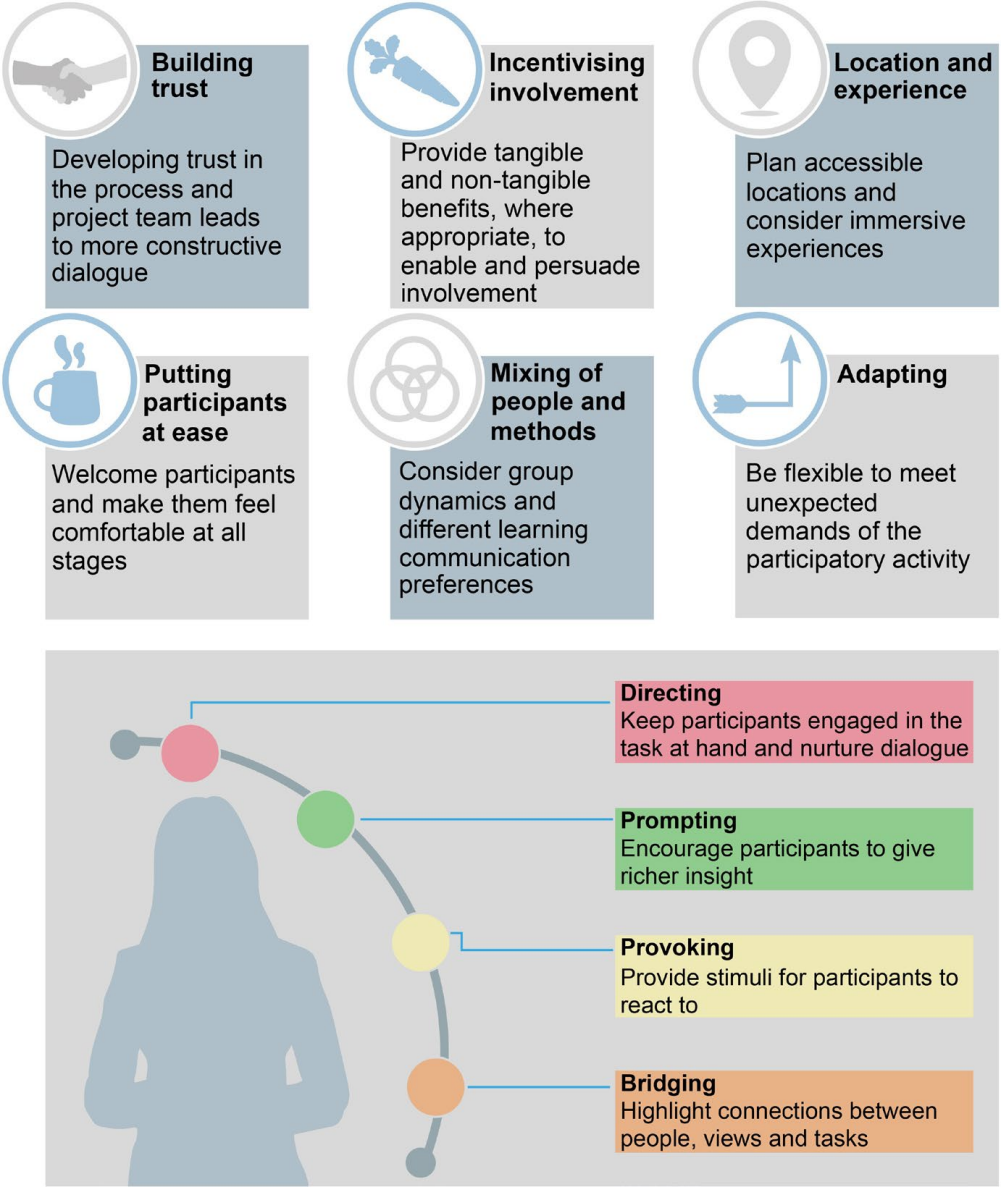
Processes and facilitation techniques for supporting high‐quality data collection in participatory activities. The six processes (top boxes) are important components, and often prerequisites, for delivering constructive participatory activities. Facilitation techniques (bottom box) can be employed by the project leader/team to guide the direction and quality of data collection

To support the features described above, we have assembled a practical toolbox of processes (Figure 3). We recognise that the processes are not mutually exclusive, either in their purpose or execution, and a particular project might not need to implement the entire suite. Nevertheless, it is important to be aware of them, and draw on them as needed.

### 3.1 Building trust

Trust is an important prerequisite for productive dialogue and should be established prior to the participatory activities beginning. Mistrust can lead to participants becoming disengaged and generating data/insights that are misleading or incomplete. Project leaders/teams can develop trust with participants by building rapport, not passing judgement, and being transparent about the project objectives and not overselling them. The time required to build trust will depend on how contentious the topic is, whether personal or sensitive information will be discussed and if responses will remain anonymous. Having an existing relationship with participants can, in some cases, be beneficial. The project leader/team may, therefore, wish to involve facilitators who are already known to the participants and are trusted. This can then lead to the transfer of trust to the wider project team. Alternatively, when exploring sensitive or other contentious issues (e.g. conflict over land management practices), facilitation from a third party who is considered to be independent and neutral may be preferable (Caribbean Natural Resources Institute, 2011).

### 3.2 Incentivising involvement

Incentives can foster inclusivity and retain participants in longitudinal projects (e.g. participatory activities with the same cohort of individuals carried out before and after a management intervention). They can entice participants to attend and to contribute, and enable the attendance of those who would otherwise have faced difficulty due to financial or other practical barriers. This can provide a better representation of desired participant groups and increase breadth. Incentives are often tangible benefits, including financial compensation for a participant's time, pre‐organised travel, childcare, food and accommodation. However, many environmental and conservation participatory projects are limited by constrained budgets. Therefore, non‐tangible incentives, such as hosting activities in a desirable venue, providing opportunities for convivial social interaction or an opportunity to contribute to (and learn about) a research project can be used to good effect. Nonetheless, it is important to be aware that providing tangible benefits can also be subject to risks. For instance, such incentives might attract individuals who simply attend to gain the incentive, rather than with the intention of participating meaningfully in the activities (Climate & Development Knowledge Network, 2014). In some cases (e.g. with policy‐makers, certain areas of health research), providing monetary incentives may also be considered unethical (Mduluza et al., 2013). Thus, project leaders/teams should carefully evaluate the most appropriate form and level of incentive for their activities and, where tangible benefits are used, mitigate risks. For example, the distribution of financial incentives can be staggered or delayed until after the activities as a way to underpin and reinforce engagement.

### 3.3 Location and experience

The setting and location for activities need to be accessible to participants, not simply wherever is most convenient to the project leader/team. The setting and location will influence the way in which people experience the participatory activities which, in turn, can affect their level of engagement. An immersive experience in situ (e.g. a particular habitat type, a specific site) can be valuable where a shared experience of an environment is required for discussion. However, using ex situ locations (e.g. community hall/room, online) may be more suitable for a range of practical reasons (e.g. ensuring the high‐quality audio recording of discussions for transcription, travel restrictions, limiting carbon emissions).

### 3.4 Putting participants at ease

Part of successfully managing participatory activities involves creating a safe space so participants feel comfortable, allowing them to relax and focus. The team should be prepared to answer questions and help participants as needed, both before the start of and during activities. The project leader/team should provide a clear timeline of what will happen when, outline any expectations, gain informed consent and address logistical arrangements if required, such as transport and accommodation. A settling in period before the activities start is essential as it allows participants to socialise and familiarise themselves with the cohort with whom they will work. This will build an individual's confidence to interact with other participants and the project leader/team. During the activities, the participants should be reassured that all knowledge is valuable (e.g. there are no right or wrong answers) and their basic needs should be taken care of (e.g. providing refreshments and adequate breaks). Once the participatory activities have finished, further communication (e.g. sending project updates) can be beneficial, especially in cases where retaining engagement across a number of activities over time is important.

### 3.5 Mixing of people and methods

Arranging people into groups according to their differences can provoke dialogue and encourage people to think outside of their usual ways of behaving and responding. However, underlying social, cultural and political dynamics could hinder open discussions and should be approached with sensitivity. Consequently, project leaders/teams should consider whether individuals need to be grouped by particular identities and social characteristics (e.g. gender, stakeholder type) to ensure they feel comfortable enough to fully reveal nuanced knowledge, insight, experiences and/or values. Using a range of methods (e.g. photo, video or audio elicitation techniques, focus groups) can provide opportunities for individuals with varying learning/communication abilities and preferences to contribute.

### 3.6 Adapting

All participatory activities should be viewed as flexible, with the ability to be modified to meet the immediate situation. This is true even when the methodological protocol is well designed and has been thoroughly piloted. Adaptation may be needed due to unforeseen challenges with participants (e.g. personality clashes, fatigue, technical issues) or because the room for improvement has been identified whilst undertaking the activities (e.g. confusing language, acoustics in venue).

## 4 FACILITATION TECHNIQUES

We also propose a suite of general facilitation techniques (Figure 3) that can be employed to maximise participant engagement and enhance data collection. Before starting, the project leader/team should review and potentially adapt the techniques, accounting for the social and cultural context of the participatory activities, as well as the background and identity of participants. For instance, cultural norms, gender, equality and social inclusion require consideration.

### 4.1 Directing

Directing conversations will ensure participants remain focused on the activities, addressing the aims of the project, whilst also leaving room for wider productive dialogue to emerge. This may involve encouraging consideration of certain topics, or gently shifting individuals away from irrelevant or unproductive discussions. For example, this might be directing conversations away from anthropogenic attributes of an environment (e.g. litter, traffic noise, facilities) when a specific focus on biodiversity is desired.

### 4.2 Prompting

Verbally prompting participants can elicit deeper and broader data. Initial responses and reactions to questions can be superficial. Prompting questions such as ‘*what do you mean by that?’* or ‘*can you expand on that point further?*’ can encourage participants to provide additional valuable information. Prompting is also a useful tool to bring other voices into the conversation. Overly dominant participants can result in misleading data and insights; prompting can help overcome this by giving other individuals a chance to contribute.

### 4.3 Provoking

Provocations stimulate a response in people by challenging them, encouraging participants to frame their perspectives as a reaction. This technique can be useful where views are so widely held and accepted that there is little discussion or verbalisation of the reasons underpinning them. The provocations could be statements, objects, images or some other kind of sensory experience (e.g. sounds, smells). In situ settings can be used to provoke participants into thinking differently about an issue through first‐hand experience of a particular location or type of setting.

### 4.4 Bridging

Connections occur between individuals (e.g. participant dynamics) within discussion (e.g. shared views/values) and methods (e.g. transition between activities in a workshop). Discussions can be enriched by stating and emphasising these connections and encouraging participants to build on them further. For instance, by clarifying, affirming and enhancing points. Bridging aids the flow of data collection, priming participants in incremental stages that may stimulate their thought processes.

## 5 CONCLUSION

Here, we have provided a toolbox of features and processes to enhance participatory approaches, encompassing facilitation techniques and prerequisites for ensuring constructive and high‐quality dialogue. The paper is not intended to be a rule book to be followed inflexibly, but thought of as a guide, providing a series of considerations for those embarking on participatory activities, which can be implemented and adapted to the project's context to help deliver high‐quality data. We hope that this work can contribute to the growing body of literature that aims to improve the use of participatory activities to address complex environmental and conservation problems.

## CONFLICT OF INTEREST

None of the authors has a conflict of interest.

## AUTHORS' CONTRIBUTIONS

All authors conceived and wrote this paper.

## Data Availability

Not applicable.
